# Clinicopathological characteristics of HER2-low breast cancer: a retrospective study

**DOI:** 10.1038/s41598-023-39372-3

**Published:** 2023-07-31

**Authors:** Man Yang, Jiale Sun, Liqiong Liu, Xiangyi Kong, Dongcai Lin, Hong Zhou, Jidong Gao

**Affiliations:** 1 Department of Breast Surgical Oncology, National Cancer Center/National Clinical Research Center for Cancer/Cancer Hospital and Shenzhen Hospital, Chinese Academy of Medical Sciences and Peking Union Medical College, Shenzhen, 518116 China; 2 Department of Nursing, National Cancer Center/National Clinical Research Center for Cancer/Cancer Hospital and Shenzhen Hospital, Chinese Academy of Medical Sciences and Peking Union Medical College, Shenzhen, 518116 China; 3 Department of Nursing, Shenzhen Hospital, Southern Medical University, Shenzhen, 518100 Guangdong China; 4 Department of Breast Surgical Oncology, National Cancer Center/National Clinical Research Center for Cancer/Cancer Hospital, Chinese Academy of Medical Sciences and Peking Union Medical College, Beijing, 100021 China

**Keywords:** Breast cancer, Cancer therapy

## Abstract

Human Epidermal Growth Factor Receptor-2 (HER2)-negative breast cancers (BCs) contain HER2-low and HER2-zero ones. HER2-low breast cancer has been receiving wide-spread concerns as the marvelous effect of novel anti-HER2 antibody-drug conjugates, however, the characteristic remains unknown. Our aim was to explore the differences of clinicopathological indicators and survival outcomes between HER2-low and HER2-0 breast cancers. We retrospectively analyzed 501 invasive breast cancer patients with complete data on HER2 status from 2017 to 2021 in our single center, of whom 415 HER2 negative patients were included for subsequent analysis. Each cohort was further divided into hormone receptor (HR) positive and HR negative subgroup. Clinicopathological factors and survival outcomes were collected and compared between HER2-low BCs and HER2-0 BCs. HER2-low BCs was obviously higher in HR positive BCs, with 277 (90.5%) HER2-low HR positive patients, 29 (9.5%) HER2-low HR negative patients, 68 (62.4%) HER2-0 HR positive patients and 41 (37.6%) HER2-0 HR negative patients (*P* < 0.001). Significant differences between HER2-low BCs and Her2-0 BCs were observed in lymph node ratio (LNR) (mean rank, 215 vs. 188 *P* = 0.014), estrogen receptor (ER)expression (90.5% vs. 62.4% *P* < 0.001), progesterone receptor (PR) expression (84.3% vs. 56.9% *P* < 0.001), Ki-67 expression (46.4% vs. 61.5% *P* < 0.001), androgen receptor (AR) expression (68% vs. 50.5% *P* < 0.001), adjuvant chemotherapy (69% vs. 79.8% *P* = 0.03). HER2-low BCs had lower histological grade than HER2-0 BCs, with grade I–II (68.7% vs. 43.1%) and grade III (22.2% vs. 43.1%) *P* < 0.01. No statistical differences were detected between the two groups for DFS and DDFS. Our results demonstrated that HR and AR status was closely related to HER2-low breast cancers. Further exploration about survival prognosis of HER2-low breast cancer is badly needed.

## Introduction

Breast cancer (BC) is the most common and the second largest causing death cancer in women worldwide^1^. Human Epidermal Growth Factor Receptor-2 (HER2) is a tyrosine kinase receptor belonging to Human Epidermal Receptor family^2,3^. The overexpression of HER2 is often associated with higher risk of disease recurrence and worse prognosis^4,5^. However, the anti-HER2 agents, including trastuzumab, lapatinib, ado-trastuzumab emtansine (T-DM1) etc., are milestone in the treatment of BC, which dramatically improved the survival rates and prognosis in HER2-positive BC, and a phenomenal success treatment is still going on^6–8^.

In current clinical practice, the American Society of Clinical Oncology/College of American Pathologists (ASCO/CAP) guidelines recommend a dichotomous classification of HER2 status, HER2-positive (HER2 +) and HER2-negative (HER2-) BC, to instruct clinical treatment. According to the most recent update guidelines (2018), HER2-positive is defined ether by immunohistochemistry (IHC) assay 3 + , or IHC 2 + with ERBB2 amplification by fluorescence in situ hybridization (FISH). While tumors with IHC 0, 1 + , or 2 + with negative FISH are determined HER2 negative^9^.

HER2-low BC is defined as those with a HER2 IHC score of 1 + or 2 + without ERBB2 amplification in most of clinical trials. According to the latest scoring systems, HER2-low BC accounts for about 45–55% in all BCs^10^.

Recently, the binary classification of HER2 status has received challenge, on one hand, because of the distinct clinicopathological features, response to neoadjuvant chemotherapy and survival outcomes between HER2-low and HER2-zero (HER2-0, IHC score of 0) BCs^11–13^. On the other hand, results from recent clinical trials suggest that novel antibody–drug conjugates (ADCs), such as trastuzumab deruxtecan (DS-8201a) and trastuzumab duocarmazine (SYD-985), have clinical effects in HER2-positive BCs as well as in those with HER2-low BCs^14,15^. The possible reasons are high cytotoxic payload, high drug-to-antibody ratio and potent bystander killing effect^4^.

Moveover, the latest clinical trial DESTINY-Breast04 shows remarkable clinical outcomes when compared DS-8201a with traditional therapy, with median PFS 10.1 months to 5.4 months and OS 23.9 months to 17.5 months in hormone receptor-positive cohort, respectively^16^. Therefore, these results reveal that HER2-low BC should be regarded as a new entity.

However, much less evidence is available regarding HER2-low BC and clinical data about the difference between HER2-low and HER2-0 are inconsistent. Meanwhile, with the substantial proportion of HER2-low BC entity, it will be meaningful to make efforts to improve survival of the entity. This demands more evidence of the clinicopathological characteristics of HER2-low BCs. We conduct this study to investigate the difference of clinicopathological characteristics between HER2-low and HER2-0 BC by retrospectively analyzing data from our single center.

## Methods

### Patient and variables selection

**Figure 1:**
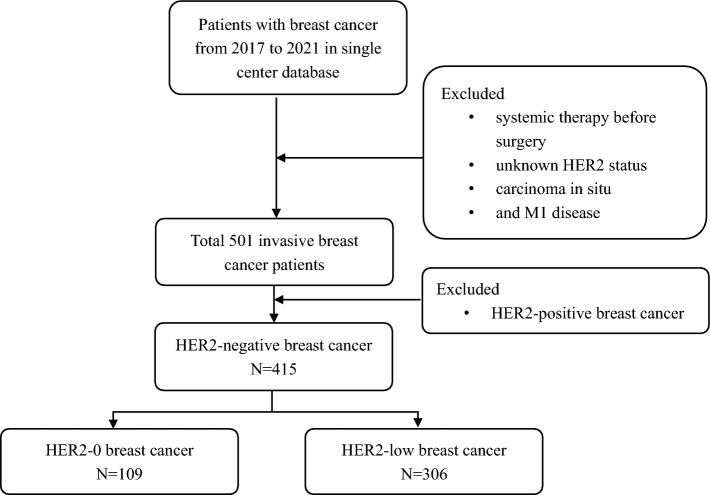
Flow chat of selecting patients in the study.

Our single center retrospective study recruited newly diagnosed female BC patients who were treated in our center from January 2017 to December 2021. Patients with systemic therapy before surgery, unknown HER2 status, carcinoma in situ, and M1 disease were excluded. Patient selection process is generated in Fig. 1. The following clinicopathological factors were collected: basic demographic characteristics [including age, body mass index (BMI), pregnancy history and menopausal status], family history of BC or ovarian cancer, tumor location, types of breast and axillary surgery, pathology data [histologic grade, lympho-vascular invasion (LVI), tumor size, number of positive lymph nodes (LNs), total number of LNs, lymph node ratio (LNR, defined as positive LNs divided by total number of LNs), pathological TNM stage (AJCC 8th edition), estrogen receptor(ER) status, progesterone receptor(PR) status, HER2 expression, Ki-67 staining, androgen receptor (AR), adjuvant chemotherapy (CT), and adjuvant radiotherapy(RT)]. This study was in accordance with the ethical principles of the declaration of Helsinki, and approved by the hospital ethics committee.

### Definition of variables and study cohort

Hematoxylin–eosin (HE) staining was used to observe breast tissue and streptavidin-perosidase (SP) staining was used to test ER, PR, HER2, Ki67, AR and LVI. Immunohistochemistry (IHC) was conducted by Roche BenchMark ULTRA (serial number 315802) and staining ≥ 1% defined as positive for ER, PR and AR. ER positive defined as HR positive in this study. The whole population was divided into four groups based on the HR and HER2 status. Antibody from Roche Diagnostics was used to detected ER, PR, HER2 and Ki67, while Maxim Biotechnologies antibody to LVI. Disease-free survival (DFS) was defined as the time from surgery to the time of first breast cancer recurrence or death from breast cancer. Distant disease-free survival (DDFS) was defined as the time from surgery to the time of distant recurrence, such as bone, lung, liver, brain, or death from breast cancer.

### Statistical analysis

Categorical variables were presented as proportions and compared using the chi-square and Fisher’s exact tests. Continuous variables were evaluated using Mann Whitney test for non-normal variables. Survival outcomes were computed using Kaplan–Meier curves, and the log-rank test was used to identify differences in DFS and DDFS rates of groups.

SPSS software (v. 26; SPSS, Chicago, IL, USA) was used to conduct statistical analysis and *P* < 0.05 was considered statistically significant.

### Ethics Statement

The studies involving human participants were reviewed and approved by the Ethics Committee of Cancer Hospital & Shenzhen Hospital, Chinese Academy of Medical Sciences. The patients provided their written informed consent to participate in this study.

## Results

**Figure 2:**
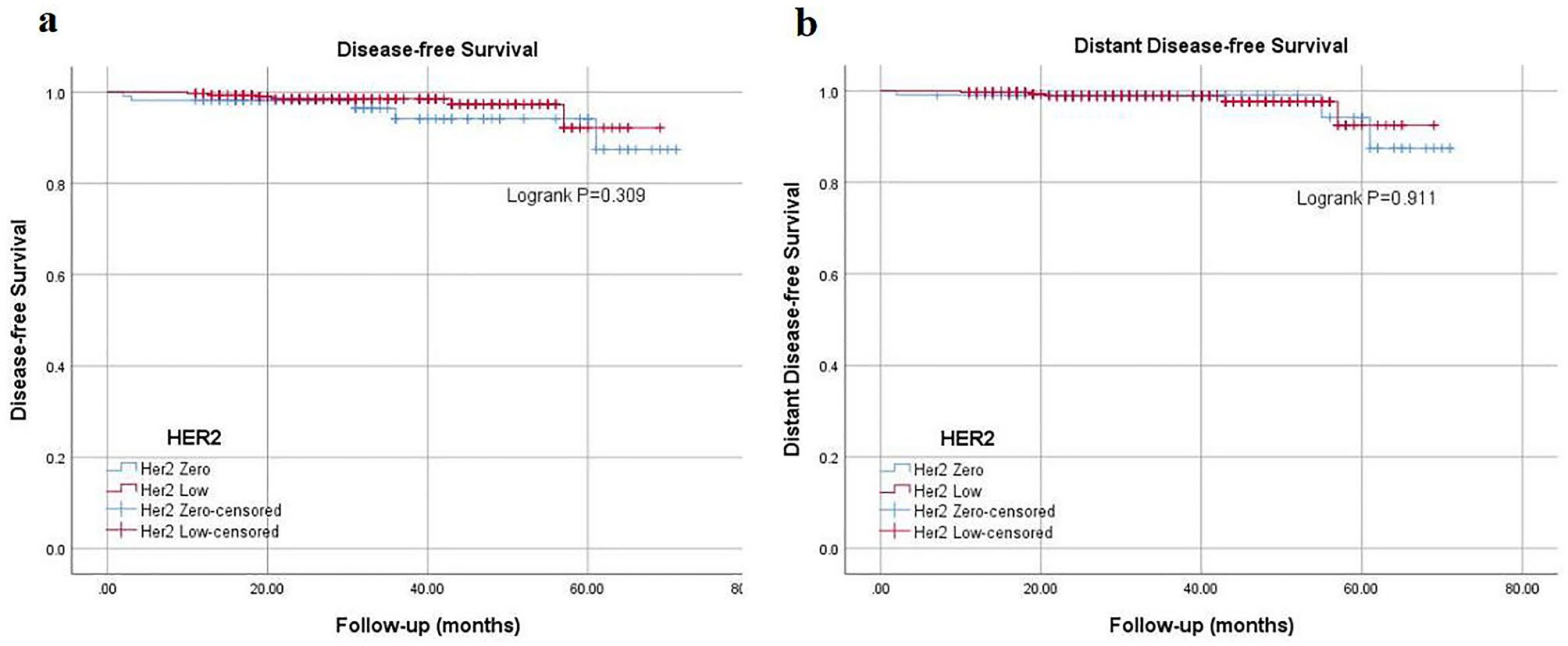
Survival outcomes of HER2-low and HER2-0 BCs.

The case enrollment and screening process are shown in Fig. 1. A total of 501 BC patients were screened, 415 (82.8%) patients were HER2-negative and included in our study, of whom 306 (61.1%) HER2-low BCs and 109 (21.7%) HER2-0 BCs. Baseline clinicopathological characteristics according to HER2 status are summarized in Tables 1 and 2. Survival outcomes of HER2-low and HER2-0 are presented in Fig. 2.

**Table 1:** Clinicopathological characteristics based on HER2 status.

Variables	HER2-0		HER2-low		*P* value
Age					0.831
≤ 50	59	(54.10%)	162	(52.90%)	
> 50	50	(45.90%)	144	(47.10%)	
Family history					0.291
No	98	(89.90%)	263	(85.90%)	
Yes	11	(10.10%)	43	(14.10%)	
Pregnancy history					0.272
No	1	(0.90%)	11	(3.60%)	
Yes	108	(99.10%)	295	(96.40%)	
Menopausal status					0.356
Premenopausal	64	(58.70%)	164	(53.60%)	
Postmenopausal	45	(41.30%)	142	(46.40%)	
BMI					0.977
≤ 25	75	(68.80%)	211	(69.00%)	
> 25	34	(31.20%)	95	(31.00%)	
Tumor location					0.438
Left	56	(51.40%)	136	(44.40%)	
Right	52	(47.70%)	164	(53.60%)	
Both	1	(0.90%)	6	(2.00%)	
Axillary surgery					0.086
SLNB	81	(74.30%)	200	(65.40%)	
ALND	28	(25.70%)	106	(34.60%)	
Breast surgery					0.993
BCS	46	(42.20%)	129	(42.20%)	
TM	63	(57.80%)	177	(57.80%)	
Histological grade					0
I	6	(5.50%)	28	(9.20%)	
II	41	(37.60%)	182	(59.50%)	
III	47	(43.10%)	68	(22.20%)	
Unknown	15	(13.80%)	28	(9.20%)	
LVI					0.403
No	74	(67.90%)	198	(64.70%)	
Yes	32	(29.40%)	104	(34.00%)	
Unknown	3	(2.80%)	4	(1.30%)	
T					0.336
T1	53	(48.60%)	169	(55.20%)	
T2	53	(48.60%)	125	(40.80%)	
T3	3	(2.80%)	7	(2.30%)	
T4	0	(0.00%)	5	(1.60%)	
N					0.063
N0	84	(77.10%)	192	(62.70%)	
N1	16	(14.70%)	74	(24.20%)	
N2	7	(6.40%)	29	(9.50%)	
N3	2	(1.80%)	11	(3.60%)	
LNR (Rank mean)	188		215		0.014
TNM stage					0.197
IA	46	(42.20%)	131	(42.80%)	
IIA	41	(37.60%)	80	(26.10%)	
IIB	11	(10.10%)	50	(16.30%)	
IIIA	6	(5.50%)	28	(9.20%)	
IIIB	2	(1.80%)	5	(1.60%)	
IIIC	3	(2.80%)	12	(3.90%)	
ER					0
Negative	41	(37.60%)	29	(9.50%)	
Positive	68	(62.40%)	277	(90.50%)	
PR					0
Negative	47	(43.10%)	48	(15.70%)	
Positive	62	(56.90%)	258	(84.30%)	
Ki67 (%)					0.007
≤ 20%	42	(38.50%)	164	(53.60%)	
> 20%	67	(61.50%)	142	(46.40%)	
AR					0
Negative	17	(15.60%)	13	(4.20%)	
Positive	55	(50.50%)	208	(68.00%)	
Unknown	37	(33.90%)	85	(27.80%)	
Adjuvant CT					0.03
No	22	(20.20%)	95	(31.00%)	
Yes	87	(79.80%)	211	(69.00%)	
Adjuvant RT					0.135
No	56	(51.40%)	124	(40.50%)	
Yes	50	(45.90%)	170	(55.60%)	
Unknown	3	(2.80%)	12	(3.90%)	

HER2, Human Epidermal Growth Factor Receptor-2; BMI, body mass index; SLNB, sentinel lymph node biopsy; ALND, axillary lymph node dissection; BCS, breast conservation surgery; TM, total mastectomy; LVI, lympho-vascular invasion; LNR, lymph node ratio; PR, progesterone receptor; AR, androgen receptor; CT, chemotherapy; RT, radiotherapy.

**Table 2:** Clinicopathological Characteristics Paired by HR Status.

Variables	HR-positive				*P* value	HR-negative				*P* value
	HER2-0		HER2-low			HER2-0		HER2-low		
Age					0.897					0.994
≤ 50	35	(51.50%)	145	(52.30%)		24	(58.50%)	17	(58.60%)	
> 50	33	(48.50%)	132	(47.70%)		17	(41.50%)	12	(41.40%)	
Family history					0.105					0.455
No	63	(92.60%)	236	(85.20%)		35	(85.40%)	27	(93.10%)	
Yes	5	(7.40%)	41	(14.80%)		6	(14.60%)	2	(6.90%)	
Pregnancy history					0.607					0.414
No	1	(1.50%)	10	(3.60%)		0	(0.00%)	1	(3.40%)	
Yes	67	(98.50%)	267	(96.40%)		41	(100.00%)	28	(96.60%)	
Menopausal status					0.597					0.441
Premenopausal	39	(57.40%)	149	(53.80%)		25	(61.00%)	15	(51.70%)	
Postmenopausal	29	(42.60%)	128	(46.20%)		16	(39.00%)	14	(48.30%)	
BMI					0.192					0.291
≤ 25	41	(60.30%)	190	(68.60%)		34	(82.90%)	21	(72.40%)	
> 25	27	(39.70%)	87	(31.40%)		7	(17.10%)	8	(27.60%)	
Tumor location					0.096					0.627
Left	40	(58.80%)	123	(44.40%)		16	(39.00%)	13	(44.80%)	
Right	27	(39.70%)	148	(53.40%)		25	(61.00%)	16	(55.20%)	
Both	1	(1.50%)	6	(2.20%)		41	(100.00%)	29	(100.00%)	
Axillary surgery					0.224					0.83
SLNB	49	(72.10%)	178	(64.30%)		32	(78.00%)	22	(75.90%)	
ALND	19	(27.90%)	99	(35.70%)		9	(22.00%)	7	(24.10%)	
Breast surgery					0.665					0.484
BCS	27	(39.70%)	118	(42.60%)		19	(46.30%)	11	(37.90%)	
TM	41	(60.30%)	159	(57.40%)		22	(53.70%)	18	(62.10%)	
Histological grade					0.068					0.409
I	6	(8.80%)	26	(9.40%)		0	(0.00%)	2	(6.90%)	
II	33	(48.50%)	177	(63.90%)		8	(19.50%)	5	(17.20%)	
III	18	(26.50%)	50	(18.10%)		29	(70.70%)	18	(62.10%)	
Unknown	11	(16.20%)	24	(8.70%)		4	(9.80%)	4	(13.80%)	
LVI					0.087					0.591
No	47	(69.10%)	179	(64.60%)		27	(65.90%)	19	(65.50%)	
Yes	18	(26.50%)	95	(34.30%)		14	(34.10%)	9	(31.00%)	
Unknown	3	(4.40%)	3	(1.10%)		0	(0.00%)	1	(3.40%)	
T					0.764					0.818
T1	36	(52.90%)	157	(56.70%)		17	(41.50%)	12	(41.40%)	
T2	30	(44.10%)	109	(39.40%)		23	(56.10%)	16	(55.20%)	
T3	2	(2.90%)	7	(2.50%)		1	(2.40%)	0	(0.00%)	
T4	0	(0.00%)	4	(1.40%)		0	(0.00%)	1	(3.40%)	
N					0.175					0.483
N0	50	(73.50%)	169	(61.00%)		34	(82.90%)	23	(79.30%)	
N1	13	(19.10%)	69	(24.90%)		3	(7.30%)	5	(17.20%)	
N2	5	(7.40%)	28	(10.10%)		2	(4.90%)	1	(3.40%)	
N3	0	(0.00%)	11	(4.00%)		2	(4.90%)	0	(0.00%)	
LNR (Rank mean)	155		177		0.058	35.4		35.6		0.966
TNM stage					0.527					0.962
IA	31	(45.60%)	120	(43.30%)		15	(36.60%)	11	(37.90%)	
IIA	23	(33.80%)	68	(24.50%)		18	(43.90%)	12	(41.40%)	
IIB	7	(10.30%)	45	(16.20%)		4	(9.80%)	5	(17.20%)	
IIIA	5	(7.40%)	28	(10.10%)		1	(2.40%)	0	(0.00%)	
IIIB	1	(1.50%)	5	(1.80%)		1	(2.40%)	0	(0.00%)	
IIIC	1	(1.50%)	11	(4.00%)		2	(4.90%)	1	(3.40%)	
PR					0.463					0.566
Negative	7	(10.30%)	21	(7.60%)		40	(97.60%)	27	(93.10%)	
Positive	61	(89.70%)	256	(92.40%)		1	(2.40%)	2	(6.90%)	
Ki67 (%)					0.506					1
≤ 20%	36	(52.90%)	159	(57.40%)		6	(14.60%)	5	(17.20%)	
> 20%	32	(47.10%)	118	(42.60%)		35	(85.40%)	24	(82.80%)	
AR					0.381					0.524
Negative	2	(2.90%)	6	(2.20%)		15	(36.60%)	7	(24.10%)	
Positive	43	(63.20%)	197	(71.10%)		12	(29.30%)	11	(37.90%)	
Unknown	23	(33.80%)	74	(26.70%)		14	(34.10%)	11	(37.90%)	
Adjuvant CT					0.893					0.067
No	22	(32.40%)	92	(33.20%)		0	(0.00%)	3	(10.30%)	
Yes	46	(67.60%)	185	(66.80%)		41	(100.00%)	26	(89.70%)	
Adjuvant RT					0.041					0.689
No	38	(55.90%)	108	(39.00%)		18	(43.90%)	16	(55.20%)	
Yes	28	(41.20%)	157	(56.70%)		22	(53.70%)	13	(44.80%)	
Unknown	2	(2.90%)	12	(4.30%)		1	(2.40%)	0	(0.00%)	

HR, hormone receptor; HER2, Human Epidermal Growth Factor Receptor-2; BMI, body mass index; SLNB, sentinel lymph node biopsy; ALND, axillary lymph node dissection; BCS, breast conservation surgery; TM, total mastectomy; LVI, lympho-vascular invasion; LNR, lymph node ratio; PR, progesterone receptor; AR, androgen receptor; CT, chemotherapy; RT, radiotherapy.

### Clinicopathological characteristics upon HER2 status

Of the 415 BCs we analyzed, 345 (83.1%) patients were hormone receptor (HR)-positive and 70 (16.9%) were triple-negative BC (TNBC). The proportion of HER2-low in HR-positive BCs was obviously higher than in TNBC: 277 (90.5%) versus 29 (9.5%). Significant differences between HER2-low BCs and Her2-0 BCs were observed in LNR (rank mean, 215 vs. 188 *P* = 0.014), ER expression (90.5% vs. 62.4% *P* < 0.001), PR expression (84.3% vs. 56.9% *P* < 0.001), Ki-67 expression (46.4% vs. 61.5% *P* < 0.001), AR expression (68% vs. 50.5% *P* < 0.001), adjuvant CT (69% vs. 79.8% *P* = 0.03). HER2-low BCs had lower histological grade than HER2-0 BCs, with grade I–II (68.7% vs. 43.1%) and grade III (22.2% vs. 43.1%) *P* < 0.01. No difference was noted between HER2-0 group and HER2-low group in age, family history, pregnancy history, menopausal status, BMI, tumor location, axillary surgery type, breast surgery type, LVI, T stage, N stage, TNM stage and adjuvant RT. However, Her2-low BCs had a tendency of having more axillary lymph node dissection (ALND, 34.6% vs. 25.7%), LVI (34% vs. 29.4%), adjuvant RT (55.6% vs. 45.9%), while less N0 stage (62.7% vs. 77.1%).

### Clinicopathological factors stratified by HR status

The prevalence of SLNB was lower for HER2-low BCs than HER2-0 BCs (64.3% vs. 72.1%) in HR-positive group, while almost equaled in HR-negative group (75.9% vs. 78%). Histological grade exhibited no statistical significance between HER2-low BCs and HER2-0 BCs when paired by HR status, but the percentage of grade I-II was higher for HER2-low BCs than HER2-0 BCs (73.3% vs. 57.3%) while the percentage of grade III was lower (18.1% vs. 26.5%) in HR-positive cohort. LNR had no significant differences between HER2-low BCs and HER2-0 BCs when stratified by HR status. The tendency of LVI was more frequent in HER2-low BCs than HER2-0 BCs (34.4% vs. 26.5%) in HR-positive group, nevertheless, with opposite result in HR-negative group (34.1% vs. 31%). The proportion of N0 stage was higher for HER2-0 BCs than HER2-low BCs (73.5% vs. 61%) in HR-positive group and (82.9% vs. 79.3%) in HR-negative group. However, all clinicopathological features mentioned above had no statistical significance between HER2-low BCs and Her2-0 BCs, irrespective of HR status, as well as the factors of tumor location, breast surgery type, T stage, TNM stage, PR, Ki-67 and AR.

### HER2-low stratified by treatment factors

The proportion of receiving adjuvant CT for HER2-0 BCs was a little higher than HER2-low BCs (100% vs. 89.7%) in HR-negative cohort, while nearly equaled in HR-positive cohort (67.6% vs. 66.8%). More patients received adjuvant RT for HER2-low BCs than HER2-0 BCs (56.7% vs. 41.2% *P* = 0.041) in HR-positive group, while opposite results in HR-negative group (44.8% vs. 53.7%).

### Survival outcomes of HER2-low and HER2-0 BCs

The median follow-up duration was 31 months. 5 patients had recurrence or metastasis in HER2-0 group and 6 patients in HER2-low group. The 5-year DFS rates for HER2-0 and HER2-low BCs were 94.1% and 92.1%, respectively. The 5-year DDFS rates for HER2-0 and HER2-low BCs were 94.1% and 92.5%. No difference was observed between HER2-0 and HER2-low group neither DFS (*P* = 0.309) or DDFS (*P* = 0.911).

## Discussion

The key novelty of the present study lies in that comparison between HER2-low BCs and HER2-0 BCs was made to explore the clinicopathological features and survival of the focused issue-HER2-low BCs, which possesses huge therapeutic potential in future clinical practice. In our current clinical practice, the dichotomous of HER2 status according to ASCO/CAP is the standard guideline, based on which the oncologists determine whether the patients should receive anti-HER2 therapy or not.

Previous clinical trial demonstrated that the addition of trastuzumab to adjuvant CT in HER2-low BCs observed no beneficial of iDFS^17^, as a result, patients with HER2 negative (HER2-0 and HER2-low) were not recommended to receive anti-HER2 therapy in current guidelines. However, the recent clinical research results has revealed a new therapeutic scenario by demonstrating potent activity of novel ADCs in HER2-low BC^14,15^. Moreover, the latest research findings of phase 3 clinical trial (DESTINY-Breast04), with 557 HER2-low BCs randomly assigned to receive trastuzumab deruxtecan or chemotherapy in a 2:1 ratio, showed significantly longer median progression-free survival (10.1 months vs. 5.4 months, HR = 0.51, *P* < 0.001) and overall survival (23.9 months vs. 17.5 months, HR = 0.64, *P* = 0.003) in trastuzumab deruxtecan cohort than the physician’s choice of chemotherapy cohort in HR positive HER2-low BCs^16^. As a result, the binary classification of HER2 status is undergone a paradigm shift, HER2-low BCs as a new entity receives widespread attention. Our work, based on single center data, supports this kind of view as well and partly elucidates the clinicopathological features and survival of HER2-low BCs.

In this study, HER2-low BCs accounted for 61.1% of all tumors, with the vast majority (90.5%) being HR positive and 9.5% being TNBC. The results were relatively consistent with previous reports^10,12,13^. The main finding of the results is that HER2-low breast cancer was closely associated with higher HR and AR expression and lower Ki67 index. As for survival outcomes, no difference was observed between HER2-0 and HER2-low group neither DFS or DDFS.

A pooled analysis from four prospective, neoadjuvant clinical trials with 2310 patients demonstrated that HER2-low BCs were more frequent in HR positive, more grade I and II, lower Ki-67 labeling index. Compared with HER2-0 BCs, lower pathological complete response rate to neoadjuvant chemotherapy was observed in HER2-low and HER2-low/HR-positive but not TNBC^11^. Similarly, Schettini et al.^13^. retrospectively reported clinical characteristics and prognostic outcomes from PAM50 gene expression data of 3689 patients with HER2-negative BC. They concluded that HER2-low BCs were associated with HR positive, older patients, male, more lymph-node involvement, but not specific prognostic implications. Furthermore, Hye Sung Won et al.^18^ suggested that HR status played an important role in HER2-low BC. Clinicopathological features of HER2-low BCs displayed differently according to HR status. HER2-low BCs were related to fewer T4 tumors, higher grade and negative lymphatic invasion in HR positive BC, while older age, high LNR and positive lymphatic invasion in HR negative BC. In summary, studies about HER2-low BC hitherto generated inconsistent results, mixed findings with good and poor prognostic indicators were observed in our study as well.

Therefore, these results preliminarily demonstrate the importance of HR status in HER2-low BCs. This implication is in line with previous research that endocrine therapy resistance might exist in HER2-low BC^19^. Similarly, anti-HER2 therapy could significantly increase the expression of ER and Bcl2 in xenograft tumors, indicating a potential ER-dependent mechanism leading to anti-HER2 resistance^20^. What’s more, discrepant response rates to anti-HER2 therapy for HER2-low BC, depending on HR status, have been observed in many studies^11,14,15,21^. Previous clinical trials suggested that patients with HR positive and HER2 negative were more sensitive to endocrine therapy than HR and HER2 positive^22,23^. Furthermore, the combination of endocrine therapy with anti-HER2 therapy has been showed to improve survival outcomes for HR and HER2 positive patients^24–26^. The main mechanism explaining researches above is the bidirectional cross-talk between HR and HER2 signaling pathways^27^. Enhanced signaling from HER family could activate downstream kinases, thereby activated ER and its coactivator AIB1^28^.

As is known to all, AR is highly expressed in breast cancer, even in TNBC^29^. Previous research had reported an association between AR and HER2 overexpression^30^, our results demonstrated that AR expression was more frequently in HER2-low than HER2-0 BCs, which was in line with other research^31,32^. Furthermore, AR targeting strategies have been applied to clinical therapy in breast cancer. A recent phase II trial combining AR inhibitor, enzalutamide, and trastuzumab in patients with HER2 and AR positive metastatic breast cancer demonstrated favorable clinical benefit rate at 24 weeks^33^. Therefore, the association between AR and HER2-low makes it possible to conduct more clinical trials to evaluate the combination of AR inhibitor and novel anti-HER2 ADC targeted therapy.

The survival outcome of HER2 low expression was unclear at present. Ergun et al.^34^ conducted a meta-analysis which included 636,535 patients and concluded that HER2-low was associated with better DFS and OS compared with HER2-0 in early-stage breast cancer, regardless of HR status. A retrospective study^35^ analyzed 351 HER2 negative patients suggested that HER2-low patients had longer 15 years of DFS (67.5% vs. 47.3%, *P* < 0.001) and OS (75.4% vs. 66.8%, *P* = 0.009) rate than HER2-0 patients. Furthermore, a pooled analysis^11^ showed that longer 3-year DFS was statistically significant in HER2-low and TNBC but not in HER2-low/HR-positive BC. However, Tarantino et al.^36^ retrospectively collected 232 HER2 negative patients and observed no difference in survival outcomes between HER2-low and HER2-0 patients. Moreover, recent population-based research demonstrated that no significant difference was observed between HER2-low and HER2-0 cohorts in OS. 5-year BC-specific survival (BCSS) according to the pathological stage was better in HER2-low group despite of HR status. In short, survival outcomes about HER2-low were inconsistent. In our study, we found no difference of DFS and DDFS between the two groups.

This study presents several limitations. First, this was a single center and retrospective research, it was vulnerable to potential bias. Second, most of our patients were collected after the year of 2019, the follow-up time was so short that longer follow-up was essential to find out more details about prognosis outcome. But our results showed potential relationship between HER2-low BCs with relative favorable outcome, namely lower Ki67 expression which is known as a prognostic biomarker in BC^37^, further research is needed to see whether it has any other clinical implication.

## Conclusion

Our data shows the complex characteristics of HER2-low BC, to which HR and AR status is closely related. No prognostic significance was observed from HER2-low expression. More research data are warranted to conclude whether HER2-low BC is a new entity with different prognosis.

## Data Availability

The original contributions presented in the study are included in the article. Further inquiries can be directed to the corresponding author.
